# A new species of the genus *Falsoibidion* Pic (Coleoptera, Cerambycidae) from Korea

**DOI:** 10.3897/zookeys.609.8846

**Published:** 2016-08-08

**Authors:** Seunghyun Lee, Seunghwan Lee

**Affiliations:** 1 Insect Biosystematics Laboratory, Department of Agricultural Biotechnology, Seoul National University, Seoul 151-921, Republic of Korea; 2 Research Institute for Agricultural and Life Sciences, Seoul National University, Seoul 151-921, Republic of Korea

**Keywords:** New species, Cerambycidae, taxonomy, Korea

## Abstract

A new species of the genus *Falsoibidion* Pic, 1922 (Coleoptera, Cerambycidae, Cerambycinae, Callidiopini) from Korea is described. Habitus and genitalia of male and female of the new species are illustrated.

## Introduction

*Falsoibidion* Pic, 1922 (Coleoptera, Cerambycidae, Cerambycinae, Callidiopini) is composed of six species worldwide. Until now, their distributional range was only restricted to the Oriental region: *Falsoibidion encaustum* Holzschuh, 1999 and *Falsoibidion infidarium* Holzschuh, 1999 from Thailand, *Falsoibidion fasciatum* Pic, 1922 and *Falsoibidion punctuosum* Holzschuh, 2003 from Vietnam, *Falsoibidion fuscipes* Hayashi, 1979 from Malaysia, and *Falsoibidion trimaculatum* Pic, 1923 from Cambodia and Laos. Lifecycle of the genus has been poorly known, only *Carica papaya* Linnaeus was reported as a host plant of *Falsoibidion trimaculatum* (Gressitt, 1970).

In this study, a new species of the genus *Falsoibidion* from central and southern part of Korean peninsula is described. This species was reported as *Falsoibidion* sp., without species level identification (Jang et al. 2015). The new species is the first record of this genus in the Palaearcic region. Moreover, we provide the first description of the genital structures of the genus *Falsoibidion*.

## Materials and methods

A total of five specimens were used in this study. Two of them were deposited in the College of Agriculture and Life Sciences, Seoul National University, Seoul, Korea, and three of them in the College of Agriculture and Life Sciences, Chungnam National University, Daejeon, Korea. All specimens were preserved in dry conditions, while hind wings and leg muscles of some of them were extracted for future molecular study. Photographs were taken with a Canon digital camera EOS 70D, Canon MP-E 65 mm f/2.8 1–5x macro lens mounted, controlled by Cognisys Stackshot.

To examine the male and female genitalia, the specimens were relaxed in distilled water for two to four hours in room temperature. Then, the genitalia were separated from the last abdominal segment using hooked pins or forceps, without removing abdomen. Separated genitalia were put into 10% KOH solution in room temperature for four to six hours. For the illustration of genital structure, Microscope (DM 4000B, Leica Microsystem, Wetzlar, Germany) with USB digital camera (Infinity3, Lumenera Corporation, Ottawa, Ontario) was used.

Several layers of photographs were combined in Zerene Stacker 1.04 software (Zerene Systems 2014; http://www.zerenesystems.com/cms/stacker).

### Abbreviations

CN
 Chungcheongnam-do 


GB
 Gyeongsangbuk-do 


GG
 Gyeonggi-do 


SNU
 Seoul National University 


CNU
 Chungnam National University 


## Results

### Falsoibidion bipunctatum sp. n.

Taxon classificationAnimaliaColeopteraCerambycidae

http://zoobank.org/38FEEE73-373D-483A-9306-4C0B4C388C1E

#### Description.

**Figure 1.**
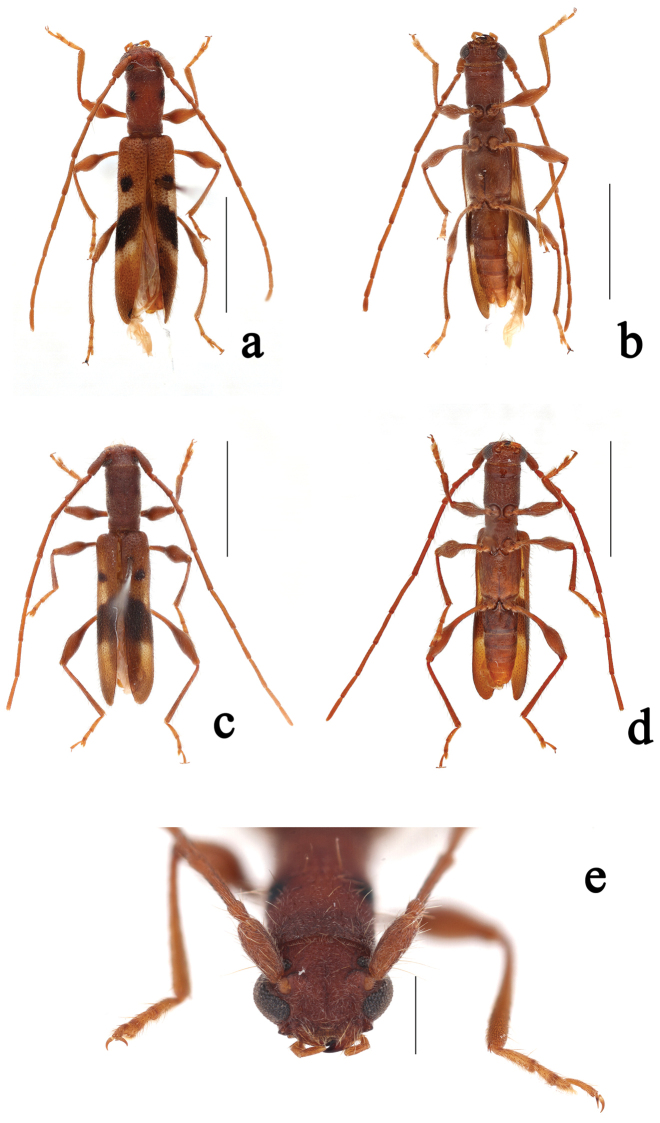
*Falsoibidion bipunctatum* sp. n. **a** Dorsal habitus, female **b** Ventral habitus, female **c** Dorsal habitus, male **d** Ventral habitus, male **e** head, female. Scale bars: **a**–**d**: 2.5 mm, **e**: 0.5 mm.

Male (Fig. 1c, d): body length: 6.32 mm, humeral width: 1.22 mm.

**Figure 2.**
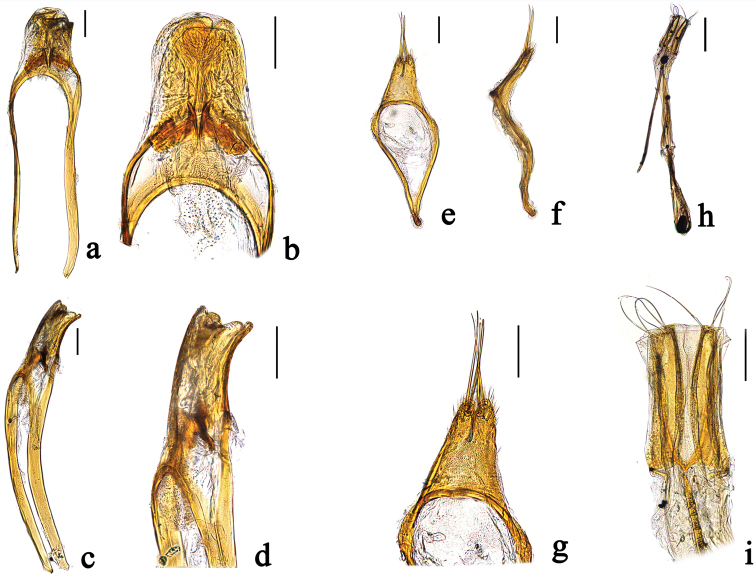
Genital structure of *Falsoibidion bipunctatum* sp. n. **a** Median lobe, dorsal **b** Median lobe, dorsal, magnified. **c.** Median lobe, lateral **d** Median lobe, lateral, magnified **e** Tegmen, dorsal **f** Tegmen, lateral **g** Tegmen, dorsal, magnified **h** sternite VIII with spiculum ventrale, dorsal **i** tergite VIII, dorsal. Scale bars: **a, b, c, d, e, f, g, i**: 0.1 mm, **h**: 0.25 mm.

Female (Fig. 2a, b, e): body length: 6.7 mm, humeral width: 1.27 mm.

Body reddish brown to dark brown with sparsely distributed long pale pubescence.

Head reddish brown to dark brown, mostly with minute sparse pubescence, some pale yellow pubescence present near the anterior margin of the labrum; frons distinctly punctured, moderately concave dorsally, some distinct tubercles present between antennal sockets; antennal sockets distinctly enlarged, distance between two sockets very short, only as long as the length of antennomere I.

Antennae 11-segmented, more or less longer than body (1:1.25 in male, 1:1.08 in female), brown, with fine short pale yellowish pubescence moderately distributed and comparatively long hairs sparsely present; antennomere I slightly swollen distally, ratio of each antennomere 1.00:0.26:0.92:0.77:1.47:1.47:1.47:1.18:1.26:0.88:1.00 in male; 1.00:0.30:0.82:0.67:1.36:1.32:1.31:1.08:1.01:0.93:0.85 in female.

Pronotum distinctly longer than wide (W/L=1.97 in male, 1.71 in female), almost rectangular in dorsal view; lateral margin weakly uneven, only finely granulose, without any distinct puncture; anterior fourth of prothorax curved downward in lateral view; teguments reddish to dark brown with a pair of distinct black spots on apical half of each lateral margin; intercoxal prosternal process narrowed toward the apex, not much developed, only reaching the posterior margin of the coxal cavities.

Elytra distinctly longer than wide (W/L=1:3.41 in male, 1:3.55 in female), almost parallel, rounded at apex, humeral margin slightly rounded, slightly more granulose than pronotum; teguments mainly brown with a small circular black spot on the basal fourth of each side of the lateral margin, a black band on the middle, forming an arrowtail-like pattern angulated toward the base, bright oval markings after this pattern;. Abdominal segments darker, with fine pale pubescence.

Leg brown, covered with moderately long pale golden hairs; hairs on tibia slightly denser than those on femur; distal third of femur largely inflated.

Male genitalia: Tegmen 0.84 mm long, 0.22 mm wide; lateral lobes with two distinct parts, gradually restricted to apex, here with fine soft hairs in dorsal and ventral side; two thick long hairs present on each lobe. Median lobe extraordinarily blunt at apex, much longer than tegmen in length, slightly curved in lateral view; median struts distinctly elongated, taking more than two third of the total length of median lobe.

Female genitalia: Sternite VIII twice as long as wide, almost rectangular, with five distinct long hairs at each side. Ovipositor missing.

#### Differential diagnosis.

The following diagnostic characters are peculiar to this species; body slender, head short, distance between compound eyes short; antennae pubescent; prothorax cylindrical, very long; femora claviform. This new species can be easily distinguished from its congeners in the following characters: two circular spot on elytra, arrowtail-like black elytral band and legs unicolor brownish.

#### Type material.

Holotype: [SNU] 1♂. Gogol-gil, Deogyang-gu, Goyang-si, GG, Korea, Light trap, 25.v.2013, D.K. Ahn.

Paratype: [SNU] 1♂. Gajang-dong, Sangju-si, GN, Korea, 9.vi.2011, street light, J.B. Choi. [CNU] 1♀. Donam-ri, Geumnam-myeon, Sejong-si, CN, Korea, 5.v.2015, J.G. Kim and H.D. Lee; 1♂, C.N.U., Gungdong-ro, Yuseong-gu, Daejeon, Korea, 9.v.2015, J.G. Kim and H.D. Lee; 1♀, same locality, 13.v.2014, Sumin Oh.

#### Etymology.

The specific epithet is named after the two black spots on pronotum and elytra.

## Supplementary Material

XML Treatment for Falsoibidion bipunctatum
